# Jefferson fracture as sport injury in weight-lifting athlete: A rare case report and literature review

**DOI:** 10.1016/j.ijscr.2024.109451

**Published:** 2024-03-05

**Authors:** Didik Librianto, Ifran Saleh, Widyastuti Srie Utami, Medisya Yasmine Librianto, Kenandi Raihan Librianto, Witantra Dhamar Hutami

**Affiliations:** a Orthopaedic Spine Surgeon, Department of Orthopaedic & Traumatology, Fatmawati Hospital, Jalan Fatmawati Raya No. 4, Cilandak, Jakarta Selatan, Jakarta 12430, Indonesia; b Orthopaedic Spine Surgeon, Department of Orthopaedic & Traumatology, Cipto Mangunkusumo National Central Hospital, Faculty of Medicine, Universitas Indonesia, Jalan Diponegoro No. 71, Jakarta Pusat, Jakarta 10430, Indonesia; c Orthopaedic Spine Surgeon, Tarakan General Hospital, Jalan Kyai Caringin No. 7, Jakarta Pusat, Jakarta 10150, Indonesia; d Faculty of Medicine, Universitas Indonesia, Jalan Salemba Raya No. 6, Jakarta Pusat, Jakarta 10430, Indonesia; e Department of Orthopaedic and Traumatology, Department of Orthopaedic & Traumatology, Cipto Mangunkusumo National Central Hospital, Faculty of Medicine, Universitas Indonesia, Jalan Diponegoro No. 71, Jakarta Pusat, Jakarta 10430, Indonesia

**Keywords:** Jefferson fracture, Sport injury, Weightlifting, Case report, Literature review

## Abstract

**Introduction and importance:**

Cervical spine fractures are rare in sports, but their potentially grave consequences mean that they must be given special attention. The aim of this study was to present the case of a recreational athlete with a fracture of C1 resulting from weightlifting.

**Case presentation:**

Young, recreational athlete came with severe neck pain right after weightlifting. There was no neurologic deficits occurred. X ray and CT scan examination showed complete fracture of the right posterior and anterior arch of C1 and disruptions of the right transverse foramen and ligament. MRI revealed no sign of impingement or compromised canal. Patient was then treated conservatively with sternal occipital mandibular immobilizer (SOMI) brace for 4 weeks. Thereafter, the neck pain resolved gradually. No neurologic deterioration occurred. At time of brace removal, patient was free of pain with normal motoric and sensory function**.**

**Clinical discussion:**

Our case was the first report of a Jefferson fracture caused by a direct injury mechanism due to the weightlifting sport. The type III Jefferson fracture produced by this contrary injury mechanism showed that with adequate force, another spectrum of injury mechanisms may be created.

**Conclusion:**

With adequate assessment and proper patient selection, Jefferson fracture can be treated effectively by SOMI brace with excellent functional outcomes.

## Highlights

• Cervical spine fractures are rare in sports, but their potentially grave consequences mean that they must be given special attention
• With adequate force, another spectrum of injury mechanisms may create C1 fracture
• With adequate assessment and proper patient selection, Jefferson fracture can be treated effectively by SOMI brace with excellent functional outcomes

## 1 Introduction

The Jefferson fracture, or fracture of the atlas, is a C1 fracture that leads to the C1 ring outward displacement [1]. The injury mechanism of the Jefferson fracture is induced by a vertical compression that loads from the occiput through the lateral part of the C1 ring [[1], [2], [3]]. The injury will cause a fracture of one or both posterior and anterior arches. Other cervical injuries commonly accompany this fracture. A potential vascular or neurologic injury could occur if the fracture is not diagnosed or treated immediately [1,2].

This type of fracture is responsible for 1—2 % of all spinal injuries and 2—13 % of acute cervical injuries. Apparently, the atlas fracture cases are dominated by male patients, with men making up 57—69 % of all the Jefferson fracture cases. Males account for up to 70 % of the cases in younger patients. However, this is not the case in elderly patients, where female patients contribute to 52 % of the cases. The distribution of the highest at-risk age range of the atlas fracture is individuals in their twenties and 80—84-year-old patients. Nonetheless, almost three-quarters of these cases happen to 50 or older patients, with 64 years of age as the mean age of diagnosis.

Jefferson fracture due to sport injury is rare. In sport injury, the Jefferson fracture most likely occurs because of a hyperextended neck, such as diving into a shallow pool, a motor vehicle accident, or a face-forward falling injury. Patients with this kind of fracture have a 0,5 % chance of suffering a vertebral artery injury (VIA) [1,4]. Only few studies reported the Jefferson fracture due to sport injury. In this study, we reported a rare case of Jefferson fracture caused by a weightlifting injury in recreational athlete. The methodology of this case report had been in line with SCARE criteria [5]. The aim of this case report was to show that the suspicion of cervical fracture should be applied in sport injury, with focus on neurological examination and how to approach the treatment.

## 2 Patient history

A 28-year-old male came to the emergency room with severe neck pain (visual analog scale – VAS of 9/10) and a right-sided headache after getting injured in a weightlifting sport thirty minutes before admission. He was doing cable crunch exercises in a commercial gym, with 40 kgs weight, accompanied by a personal trainer. He was in a kneel-down position with his trunk and abdomen flexed around 45 degrees when the machine's cable broke, causing the rope's attachment to hit his neck, and his head abruptly struck the ground.

## 3 Clinical finding

On arrival, the patient was alert and well oriented. He only complained of neck pain and headache on the right side of his head. He has been in weightlifting sports for almost two years, regularly, 5 to 6 times a week, and under personal trainer supervision. Cable crunch has been added to his exercise routine for one and a half years.

Physical examination revealed a mesomorph body shape, 176 cm of height and 91 kg of body weight, with normal vital signs. Pain on the upper right and posterior part of the neck (VAS 9/10), pain in neck flexion (VAS 8/10), and neither bruising nor neurologic deficits were found.

## 4 Timeline

**Table:** 

Time	Clinical finding	Treatment
One hour before hospital admission	Severe neck pain with right-sided headache after weight lifting	Neck immobilization and patient was brought to emergency room
One hour after injury	Severe neck pain with right-sided headache after weight lifting. Normal neurologic functions	Immobilization using SOMI brace for 4 weeks
4 weeks after injury	No complaint of pain and neurologic function	SOMI brace was released

## 5 Diagnostic assesment

**Fig. 1:**
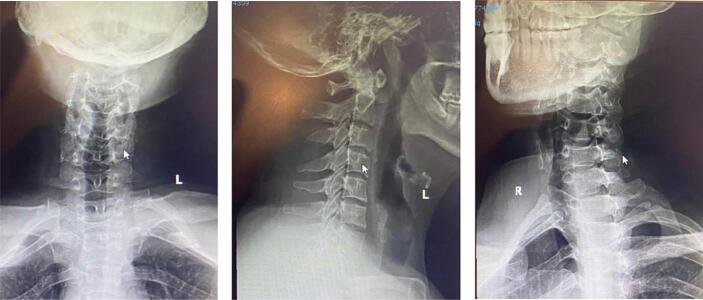
Cervical X ray. X-Ray on admission showed loss of cervical lordosis.

**Fig. 2:**
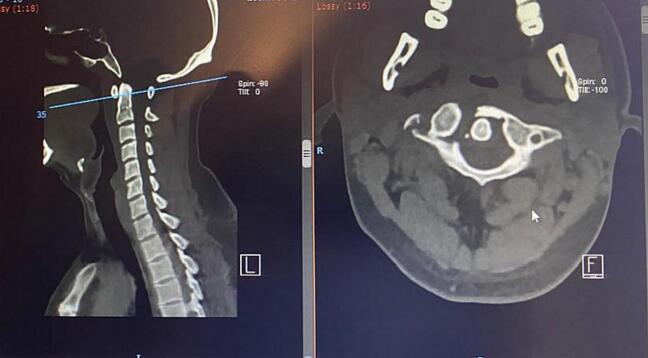
CT axial of the cervical spine. Axial CT showed complete fracture of the right posterior and anterior arch of C1 and disruptions.

Anteroposterior and lateral cervical x ray showed an unclear fracture line (Fig. 1). Axial computed tomography demonstrated a complete fracture of the right posterior and anterior arch of C1 and disruptions of the right transverse foramen and ligament (Fig. 2). MRI revealed no sign of impingement or compromised canal.

The patient was diagnosed as unstable Jefferson burst fracture type III with unilateral fracture of the right anterior & posterior rim and disruption of the right transverse foramen due to hyperextension and direct injury to the neck.

## 6 Therapeutic intervention

A SOMI brace was applied for four weeks, analgesia was prescribed, and the patient was sent home.

## 7 Follow up and outcomes

**Fig. 3:**
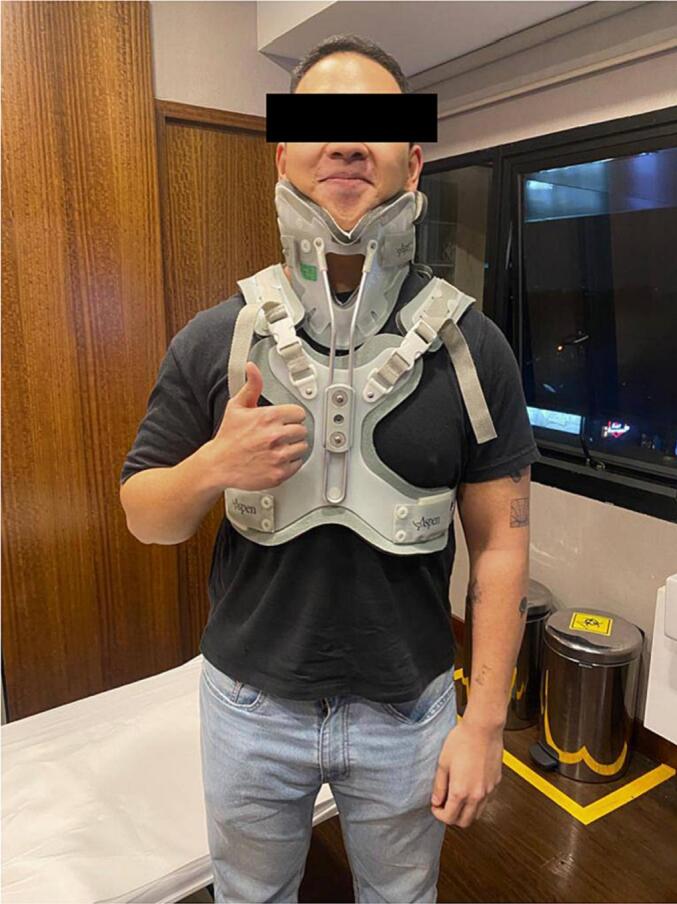
Clinical presentation at follow-up. On 4th week follow-up, the neck pain was resolved, and no neurologic deficit was found (picture was included according to patient' consent).

On 4th week follow-up, the neck pain was resolved, and no neurologic deficit was found (Fig. 3). Patient had no complaint.

## 8 Discussion

In 1920, Jefferson described a C1 fracture, which now is associated with his name, as a response to lateral spreading forces. Since then, clinical reports and laboratory tests have been done to confirm the mechanism of this fracture. [6] Oda T et al. and Hays MB et al. confirmed Jefferson's theory of axial load to the ring of C1 by reproducing the fracture in the laboratory [7,8]. Other authors reported cases of three- and four-part C1 fractures by the same axial load mechanism [3,[8], [9], [10]]. Nevertheless, uncertainty about the axial force theory being the only mechanism creating the Jefferson fracture is still doubtful among authors. Chang D et al. described a three-part Jefferson fracture caused by a rear-end collision car accident. [11] A pedestrian injury and odontoid process breaking through the anterior ring was also reported to create a Jefferson fracture [1,10]. These studies showed other biomechanical points of view and added more spectrum of injury to the fracture.

Related to the mechanism of injury, the amount of force needed to create the fracture has also been discussed in several studies. Panjabi et al. were able to produce C1 burst fractures in an axially loaded specimen with an average force of 3050 N. An impact loading as low as 1900 N was also found to create burst fractures, even though pressures as high as 3200 N did not create a fracture in 5 of their specimens [7]. Beckner et al. found a mean considerable force needed to create the C1 ring fracture was around 2288 N. [6] Stephen Smith was the first surgeon to investigate the strength of the atlas in 1871. He was able to break the isolated anterior arch by applying 308 lbs.

Our case has some unusual features. Similar to Chang D et al. [11], the mechanism of injury producing the fracture in our case didn't correspond to the axial load mechanism first described by Jefferson. Yet, the same fracture pattern was found. With 40 kg weight, a single fixed pulley creates around 4000 N effort force. This effort force was more significant than the mean force reported by several studies. For these reasons, a direct injury to the posterosuperior part of the neck with enough power does not preclude the possibility of a C1 fracture.

According to a rule proposed by Spence et al., lateral mass displacement of C1 less than 7 mm was considered stable and can be treated effectively by external immobilization [10,12,13]. Landells et al. [14] suggest that operative treatment is only performed when instability persists after six weeks of external immobilization. The halo vest was the traditional type of cervical immobilization used in treating Jefferson fractures. [14,15] In more recent studies, Minerva jackets and rigid cervical collars have also been used in stable fractures [16]. Currently, no standard conservative treatment method has been approved. Although considered the gold standard for cervical immobilization, the halo vest has been associated with complications such as infections, CSF leaks, and brain injury [[17], [18], [19], [20]]. Conservative treatment used in our case, a SOMI brace, improved the patient's pain within two weeks. Follow-up radiographs showed no sign of instability. The result of this case is in line with a study by Lee et al., who treated 12 patients with stable Jefferson fractures with a rigid cervical collar. The difference is Lee's patients took 10 to 12 weeks to achieve clinical improvement [21].

The clinical implication of this report was that the treatment options for the Jefferson fracture vary depending on the presence of other cervical injuries related to the main injury. [1,14,22,23] For a nonoperative treatment, the use of a halo-thoracic brace, rigid collar, or sterno-occipitomandibular immobilization is effective for a stable fracture. [1,2,6,14] Meanwhile, operative treatment is used for more unstable and complex fracture cases [22]. More importantly, this report showed that sport injury can cause cervical injury, therefore complete neurologic examination should be performed cautiously.

## 9 Conclusion

In conclusion, our case is the first report of a Jefferson fracture caused by a direct injury mechanism due to the weightlifting sport. The type III Jefferson fracture produced by this contrary injury mechanism showed that with adequate force, another spectrum of injury mechanisms may be created. Yet, the correlation between the patient's high muscle mass and its effect on the severity of injury is still unknown. Furthermore, the treatment chosen in our case, the SOMI brace, compared to a rigid cervical collar, was associated with a shorter healing time. Other benefits of a rigid cervical collar are supposedly also found in using a SOMI brace.

## Consent

Written informed consent was obtained from the patient for publication of this case report and accompanying images. A copy of the written consent is available for review by the Editor-in-Chief of this journal on request.

## Ethical approval

The ethical approval was not required for this case report.

## Funding

The authors received no financial support for the research, authorship, and/or publication of this article.

## Author contribution

Didik Librianto: study concept, data collection, data interpretation, and writing the paper.

Ifran Saleh: data collection, data interpretation and writing the paper.

Widyastuti Srie Utami: data collection, data interpretation and writing the paper.

Medisya Yasmine Librianto: data collection, data interpretation and writing the paper.

Kenandi Raihan Librianto: data collection, data interpretation and writing the paper.

Witantra Dhamar Hutami: data collection, data interpretation and writing the paper.

## Guarantor

Didik Librianto.

## Research registration number

This case report is not a first in man study.

## Conflict of interest statement

The authors certify that They have NO affiliations with or involvement in any organization or entity with any financial interest or non-financial interest in the subject matter or materials discussed in this manuscript.
